# Sustained Local Delivery of Structurally Diverse HIV-1 Microbicides Released from Sublimation Enthalpy Controlled Matrices

**DOI:** 10.1007/s11095-012-0811-8

**Published:** 2012-06-27

**Authors:** Simi Gunaseelan, Philippe A. Gallay, Michael D. Bobardt, Charlene S. Dezzutti, Timothy Esch, Richard Maskiewicz

**Affiliations:** 1 Department of Pharmaceutical Sciences, School of Pharmacy Loma Linda University, 11175 Campus Street, Chan Shun Pavilion 21018, Loma Linda, California 92350 USA; 2 Department of Immunology and Microbial Science, IMM-9 The Scripps Research Institute, 10550 North Torrey Pines Road, La Jolla, California 92037 USA; 3 Magee-Womens Research Institute, 204 Craft Avenue, Pittsburgh, Pennsylvania 15213 USA; 4 Department of Obstetrics, Gynecology and Reproductive Sciences, University of Pittsburgh, 204 Craft Avenue, Pittsburgh, Pennsylvania 15213 USA

**Keywords:** enthalpically controlled release, HIV-1, intravaginal delivery, prolonged antiviral effect, subliming solid matrix

## Abstract

**Purpose:**

Use of coital-dependent products to prevent HIV-1 transmission has resulted in mixed success. We hypothesize that incorporation of antiviral drug candidates into a novel controlled delivery system will prolong their activity, making their use coital independent, thus increasing their chance of prophylactic success.

**Methods:**

Tenofovir, emtricitabine, and C5A peptide HIV microbicides were mechanically incorporated into matrices comprising a series of subliming solids. Matrix sublimation rates and drug release rates were measured in three *in vitro* and one *in vivo* environments intended to model human vaginal interior. Antiviral activity studies evaluating matrix incorporated microbicides were performed using *in vitro* cell cultures and human ectocervical explants.

**Results:**

Drug release rates were identical to matrix sublimation rates, and were zero order. Differences in matrix material sublimation enthalpies determined drug release and matrix erosion rates in a thermodynamically definable manner, *in vitro* and *in vivo*. Durations of release ranging from several days to several months were readily achieved. Prolonged duration of anti HIV-1 activity was shown for matrix incorporated microbicides, using ectocervical explant and cell culture models of HIV-1 infection.

**Conclusion:**

Subliming solid matrices show promise as a delivery system providing multi month intravaginal release of a wide range of HIV-1 microbicides.

## INTRODUCTION

Prevention of AIDS infection in healthy women during intercourse with HIV positive partners through use of condoms or topical applications of antiviral microbicide continues to meet with limited success for a number of personal and social reasons (1). The majority of HIV-1 microbicides delivery systems under current clinical evaluation employ coital-dependent quick release formulations administered in the form of aqueous-based gels applied shortly prior to and/or after coitus. Such delivery systems usually fail to provide inhibitory concentrations for a sufficient period of time to avert transmission that may be a result of repeated sexual activity, and the viral shedding typical of acute HIV-1 infection, and therefore require frequent and repeated microbicide application (2). Windows of opportunity for untreated viral spread and progression of infection can also occur, ultimately requiring a switch from prophylaxis to more problematic systemic therapy, using several of the same antiviral agents employed as HIV-1 microbicides. These limitations can be resolved by employing coitally independent sustained and extended duration delivery systems intended to simplify and assure infection prevention. Several potential products could ultimately utilize an intravaginal ring, which would be inserted into the vaginal cavity well before coitus and assist in the local retention of the administered dose. Sustained release of and prolonged protection by several possible microbicides would occur through the intra-ring incorporation of the drug delivery system described herein. Such sustained local delivery of microbicides should ultimately be able to ensure that inhibitory anti-HIV-1 agent concentrations are constantly available *in situ* to continuously block the early steps of sexual HIV-1 infection (2).

 Effective prophylaxis against sexually transmitted HIV-1 entails improved subject compliance through continuous and prolonged duration delivery of periodically administered HIV-1 microbicide (3,4). Continuous intravaginal release in a coitally independent manner can improve efficacy but is constrained by the drug delivery technologies which are available. The goal of this study was to identify, and perform proof-of-concept characterization of a drug delivery technology demonstrating controlled release of multiple microbicides while possessing fewer limitations than any of the currently employed delivery methods. Current problem areas with existing techniques for sustained drug release include rates of intravaginal microbicide delivery which typically decrease substantially in the course of their prolonged release (5), and rates and durations of microbicide sustained release based on hindered diffusion often being more dependent on the size, structure, and physiochemical properties of a candidate antiviral drug than on the intrinsic performance of the delivery system. This often makes achievement of specific therapeutic release rates and durations problematic, especially for high molecular weight or hydrophilic agents released from polymer monoliths (6,7). Other problems confounding attempts at providing reproducible and constant intravaginal drug release rates or durations include variability of intravaginal physiological state such as local pH (range of 3.0 to 5.5), hydration state, and flora (8–10), all of which would affect durations of release/efficacy through variability in the erosion rate of a drug-incorporating matrix/capsule where release of drug is achieved through delivery system dissolution/hydrolysis. Recent identification and early clinical evaluation of peptide and protein based HIV-1 microbicides further complicates efforts towards development of sustained release versions of novel and potentially highly selective HIV-1 prophylactic agents (11) due to their generally poor chemical and physical stability in the intravaginal milieu upon bolus administration of simple formulations and during long-duration delivery (12).

 A new process for sustained release of drugs into a defined environment, employing neither conventional drug diffusion nor matrix/membrane dissolution (13), has been identified and holds promise towards solving these problems. This process entails incorporation of drug into a matrix, and subsequent matrix erosion, but differs in that drug release is not due to either hydrolysis or dissolution of the matrix material, or due to diffusion of water into or drug out of a non-erodable matrix. Rather, release of drug from chemically inert and water insoluble matrices occur by surface erosion achieved through sublimation (direct conversion of solid to a gas) of matrix, allowing sequentially exposed drug particles to be delivered to the local environment of the administration site. The hypothesis is that a novel delivery system based on drug-loaded matrix sublimation can provide release rates independent of drug molecule size and physicochemical properties, but instead be primarily dependent on enthalpies of sublimation of specific matrix materials. A secondary hypothesis is that measures of long duration release obtained using unconventional *in vitro* models can translate into HIV microbicide activity prolongation *ex vivo*, and are reflective of sublimation (and mass transport) processes which occur intravaginally.

 Subliming solid matrices consist of hydrophobic, chemically and biologically un-reactive organic substances (14), which due to their having weak intermolecular interactions in the solid state, can directly transform into vapor at physiological temperature. Examplary molecules possessing this attribute comprise several perfluorocarbons and cyclic/branched alkanes achieving substantially spherical structural conformations. Subliming matrices chosen for demonstrating the feasible range of microbicide controlled release durations include norbornane (NOR), hexamethylethane (HME), hexamethylcyclotrisiloxane (HMCS), perfluoroundecane (PF-11), perfluorododecane (PF-12), and cyclododecane (CDD); between them possessing a sublimation enthalpy range between 40 and 76 kJ/mol (15–17).

Anti-HIV drug candidates, emtricitabine and tenofovir, which have achieved clinical confirmation of microbicidal efficacy via topical (18) and oral administration (19), were chosen to evaluate delivery kinetics from subliming matrices, along with the novel but chemically unstable peptide microbicide C5A (20). These drugs were also employed to demonstrate that sublimation based sustained release in cell and tissue cultures can extend anti-HIV-1 activity relative to the marginally prophylactic durations achieved by single bolus administration of simple aqueous formulations at a given dose. Choice of HIV-1 microbicides for evaluation of this novel drug delivery system was additionally based on their possessing significant differences in physicochemical properties, allowing the abovementioned demonstration that subliming solid matrix based control of release rate is independent of the nature of the drug substance. We evaluated release of tenofovir disoproxil fumarate (TDF) a charged but poorly soluble molecule [M.W. = 287], emtricitabine (FTC) an uncharged but highly water soluble molecule [M.W. = 247], in addition to the biotinylated version of a novel microbicide C5A (bC5A) a hydrophobic, low solubility, chemically reactive eighteen amino acid peptide [M.W. = 2,537].

 Demonstration of a broad range of controlled release rates and associated prolongations of antiviral efficacy for the above microbicide molecules incorporated within a series of subliming matrices, should provide sufficient rationale for critical follow-up studies examining the long-term intravaginal and systemic safety of the chosen subliming solid substances and their use for prolonged drug delivery. The results discussed herein will also serve as the foundation for our ultimate goal, development of robust sexual HIV prophylaxis using an intravaginal-ring-based sustained retention system, incorporating subliming matrices which will then control the rate and delivery duration of multiple microbicide drug substances.

## MATERIALS AND METHODS

### Chemicals

CDD was obtained from Kremer Gmbh (Aichstettin, Germany). PF-11 and PF-12 were obtained from Exfluor Research Corporation (Round Rock, Texas, USA). ADM, NOR, HME, and HMCS were purchased from Sigma-Aldrich (St. Louis, Missouri*,* USA). Matrix materials were further purified by resublimation at atmospheric pressure. FTC and bC5A [SWLRDIWDWICEVLSDFK-Biotin] were obtained from the laboratory of Dr. Philippe Gallay (The Scripps Research Institute, California, USA). TDF was provided by CONRAD (Arlington, Virginia, USA). HPLC grade acetonitrile was purchased from Fisher Scientific (Pittsburgh, Pennsylvania, USA). Trifluoroacetic acid and formic acid were obtained from EMD Chemicals (Gibbstown, New Jersey, USA), and Sigma-Aldrich (St. Louis, Missouri*,* USA) respectively. Nano pure water (0.2 micron) was obtained from Barnstead NANOpure Water Purification Systems (Asheville, North Carolina, USA).

### Preparation of Cylindrical Matrix Formulations Incorporating Different Drugs for Release Rate Determinations

The three drugs were micronized using mortar and pestle. Each of the powders were then homogenously dispersed into a given subliming matrix powder at 1.0 wt/wt percent loading through serial dilution in a mortar and pestle. 5 × 5 mm cylinders of drug-loaded matrices which were longitudinally enclosed in impermeable PVC sheaths (providing constant sublimation surface areas of 0.39 cm^2^ per cylinder) were prepared by weighing drug loaded powders (to yield final pellet weights of 400 mg for PF-11 and PF-12 matrices, and 175 mg for NOR, HMCS, and CDD matrices) into a steel mold, and then compressing with a force of 545 lbs transmitted by the corresponding cylindrical die for 1 s, using a Carver Hydraulic Press (Carver Inc., Wabash, Indiana, USA).

### Preparation of Placebo and Drug-Loaded Discs for *Ex Vivo* and *In Vivo* Evaluations

Test samples for *ex vivo* studies having dimensions of 5 × 5 × 3 mm, and containing 0.1% microbicide, were prepared as previously described, to yield final pellet weight of 400 mg for PF-11 and PF-12 matrices, and 175 mg for ADM, HMCS and CDD matrices. Placebo (non drug loaded) cylinders for intravaginal sublimation rate determinations were prepared in a manner identical to the one employed for cylinders and discs used for *in vitro* and *ex vivo* studies. Cylinder dimensions were 7.5 × 15 mm and yielded target pellet weights of 750 mg for NOR and HMCS based test articles.

### Matrix Sublimation and Drug Release Rate Determination

*In vitro* determinations of matrix sublimation and drug release rates were performed in a model environment intended to approximate limited hydration within the human vagina, where internal fluid volume is low (21) and surface area is relatively high (22). Sublimation mediated erosion of constant surface area matrix cylinders was therefore initiated and maintained through removal of sublimed vapor from matrix surface by defined and reproducible convection produced by air at 37°C and 100% relative humidity. This convective environment is similar in terms of mass transport to the one maintained during *ex vivo* activity prolongation studies using human ectocervical explants (see below). These two sublimation based models are defined as “accelerated” relative to the convective (sublimed vapor transport) environment present *in vivo* in ewes, and in the relatively low gaseous mass transport (partially sealed container) cell culture based efficacy studies (again see below).

Uncapped glass scintillation vials containing PVC encased subliming cylinders were withdrawn from the incubator at periodic intervals, and reinserted after cylinder gravimetry, and released drug recovery. Drug which was exposed/released after each incubation interval was recovered by rinsing each cylinder and surrounding vial surface for 30 s with 4.0 ml of a (i) 50% acetonitrile/water solution, for bC5A; and (ii) 10% acetonitrile/water solution, for FTC and TDF. All gravimetric and release data are reported as mean ± SEM (*N* = 4). Control experiments have shown that released amounts of each of the employed drug substances were completely dissolved by this procedure, without any measurable concomitant dissolution of unsublimed matrix cylinders.

### Quantitation of Released Drugs

Amounts of released bC5A, FTC, and TDF (in rinse solutions) were measured using a Waters Alliance HPLC system (CLARITY software) equipped with a UV detector and a reversed phase C_18_ column (Waters, Inertsil® ODS-2, 5 μm, 4.6 × 150 mm), quantitation being performed at 280 nm. Gradient elution of peptide microbicide employed water containing 0.1% trifluoroacetic acid (solvent A), and acetonitrile containing 0.1% formic acid (solvent B). A linear gradient (*t* = 0 min, 5 B, 95% A; *t* = 2 min, 5% B, 95% A; *t* = 15 min, 95% B, 5% A; *t* = 16 min, 5% B, 95% A; *t* = 20 min stop) employing a flow rate of 1.2 ml/min at 40°C was used. HPLC quantitation of FTC and TDF were performed at ambient temperature using an isocratic reversed phase method. 10% acetonitrile in water containing 0.1% trifluoroacetic acid and 0.1% formic acid was used as mobile phase at a flow rate of 1.0 ml/min.

### Intravaginal Matrix Sublimation in Sheep

Adult ewes (Non Pregnant Western Sheep, Nebeker Ranch, Lancaster, California, USA) were employed, per a IACUC approved protocol. Cylindrical pellets were individually inserted into the vaginas of four 100–150 lb adult ewes. Prior to insertion (one per ewe) each matrix cylinder was placed in a fine fiber coarse weave plastic netting pouch tied with suture, to facilitate recovery. Cylinders were inserted using a commercial vaginal pessary applicator, and recovered by hand. *In vivo* rates of sublimation were determined by measuring cylinder weight changes (after removal of netting) at periodic intervals. Gravimetric data are reported as mean ± SEM (*N* = 4).

### Viability of Human Ectocervical Tissue

Human ectocervical tissue was acquired through an IRB-approved protocol from surgical remainders. Polarized explants were prepared as described (23,24). One 5 × 5 × 3 mm disc was placed on the apical surface of the treated explants with 100 μl of medium on the day of set-up and cultured for 5 days. Matched controls, no treatment or exposure to nonoxynol-9 (N9), were set-up in parallel. After 5 days, the solids were removed and the explants were rinsed with HBSS. Viability was determined using the MTT [1-(4,5-dimethylthiazol-2-yl)-3,5-diphenylformazan] assay.

### Viability of Cultured Human PBMC, Macrophages and T-Lymphocytes

Cells were plated in clear-bottom 6-well plates (BD Biosciences) in the presence or absence of matrix discs for a week. Cell Quanti-MTT™ reagent (Gentaur) was added and cells incubated for 4 h at 37°C. Solubilization solution was added and plate shaken for 1 h at room temperature. *A*570 nm was measured on a SpectraMax 384 Plus reader (Molecular Devices). As control, cells were treated with 0.01% saponin for a week. No washes were performed to maintain a continuous exposure of cells to solids.

### Durations of Anti-HIV Activity in Human Ectocervical Explants

Polarized explants were set-up in duplicate and one 5 × 5 × 3 mm PF-11 disc was placed on the apical surface of the explants with 100 μl of medium on the day of set-up and cultured for 3 days, under conditions where both the tissue surface and the applied subliming matrix were in direct contact with air, which was in turn readily exchangeable with the external atmosphere. In this sense, experimental conditions for mass transport of sublimed vapor during explants studies were similar to those present during “accelerated” *in vitro* sublimation and drug release rate determinations. To model antiviral efficacy achievable by single bolus administration of the drug, 5 μM of TFV or FTC was added to the apical surface 1 h prior to the first HIV-1 exposure. Controls included untreated polarized explants, and explants in continuous contact with placebo PF-11 discs. HIV-1_BaL_ (5 × 10^4^ TCID_50_) was applied apically and all explants were cultured overnight. After culture, the explants were washed and fresh medium added to the basolateral compartment. Viral challenge was repeated three additional times (4 total HIV-1 exposures) every 3 to 4 days over 10 days. Discs remained on the apical surface until they sublimed away (typically 11 days), but were still present at cessation of challenge. Every 3 to 4 days, basolateral supernatant was collected and replenished through 21 days. The basolateral supernatant was tested for HIV-1 replication by p24gag ELISA (Perkin-Elmer, Inc. Waltham, MA). Results are expressed as pg/ml of HIV-1 p24 release over 21 days. The median ±95% confidence interval is shown. Explants were fixed in formalin at 21 day and endpoint immunohistochemistry for HIV-1 p24gag expressing cells was performed (23,24). Pink deposits indicate HIV-positive cells.

### Durations of Anti-HIV Activity in Human Macrophage and TZM Reporter Cell Cultures

Macrophages were isolated from blood donors as described previously (20) and placed in cell culture for a week for cellular differentiation into 6-well plates. Due to the need to maintain sterility, only limited mass transport (air flow between wells and external atmosphere) of sublimed vapor was possible, making this particular *in vitro* methodology more comparable to the relatively slow convective processes thought to occur intravaginally. Matrix discs or unformulated microbicides in aqueous solution were then added to wells. At various time points (week 1 to 4), macrophages were exposed to HIV-1 R5 GFP and infection analyzed after 48 h by FACS (% of GFP-positive macrophages). TZM reporter cells were placed into 6-well plates in cell culture together with discs or unformulated drugs. At various time points, supernatant aliquots were collected and stored at −20°C. After 32 days, collected aliquots were thawed and individually added to TZM reporter target cells together with HIV-1 (JR-CSF 200 pg of p24). Cells were washed after 3 h. Two days post-infection, TZM cells were lysed and betagalactosidase activity in cell lysate was quantified. Data (triplicate) are expressed in relative light units. Results are representative of three independent experiments.

## RESULTS and DISCUSSION

Sublimation of a water insoluble matrix surrounding, entrapping, and eventually exposing drug particles as a mechanism for sustained release has not previously been considered. This is perhaps due to the perceived small range of sublimation (and associated drug release) rates possible, or due to perceived toxicity of solid compounds commonly associated with sublimation (naphthalene, camphor). However, by employing matrix materials comprising volatile spherical or globular and primarily alkane based molecules, a large range of sublimation/drug release rates (days to months) are envisioned, while putatively also providing and maintaining complete biological and chemical protection of matrix contents. Herein we determined sublimation and concomitant drug release rates for several volatile matrix materials possessing enthalpies of sublimation ranging between 40 kJ/mol and 76 kJ/mol (17).

Our strategy towards collectively assessing this delivery system’s potential for sustained intravaginal drug release from subliming solid matrices, employed four models, and entailed a range of local environment (administration site) dependent convective transport rates. Relatively rapid mass transport (via convection/diffusion of sublimed vapor away from an eroding matrix surface) is required for sublimation to be continuous, and is expected to be fast relative to the intrinsic sublimation rates of studied matrix materials, in all of the models employed in this study. 1) The first model entailed “accelerated” (rapid convection) *in vitro* sublimation and drug release into moist air. This controlled mass transport procedure is designed to account for low fluid volumes present within the vagina and concomitant low intravaginal hydration state. Estimates of intravaginal hydration (at steady state) range from 0.012 to 0.04 ml of mucus per cm^2^ of intravaginal surface (21,22). This particular procedure was employed for all precise rate determinations. 2) The second model also employed rapid convection, and entailed *ex vivo* release of microbicides directly onto ectocervical tissue, to demonstrate prolonged anti-HIV-1 activity and absence of matrix material toxicity. 3) The third model employed slow convection *in vitro* release directly into cell culture medium to demonstrate prolonged (>30 days) antiviral protection (and lack of toxicity) in several cell types commonly employed for microbicide screening. 4) The final model entailed intravaginal incubations in ewes, again entailing slow convection/diffusion conditions, providing *in vivo* matrix sublimation rates, in a defined and potentially relevant to women environment.

It should be noted that classical *in vitro* release methodologies modeling systemic delivery entail immersion of formulation/device samples into an aqueous or mixed solvent “reservoir”, or “release medium”, thereby allowing either matrix erosion or intramatrix diffusion, with drug dissolution occurring during or immediately subsequent to release (13). Since exposure of matrix incorporated drug particles through intravaginal sublimation of matrix surface does not require the mediation of liquid water, the primary *in vitro* release technique employed for this study is analogous to those often employed for *in vitro* evaluation of transdermal drug delivery systems using skin samples, where no aqueous solution component is present during release rate measurements of patch incorporated drugs (25).

Intravaginal dissolution of drugs exposed on matrix surface subsequent to local sublimation should occur at rates and to extents dependent on (assuming reasonable aqueous drug solubility) degree of vaginal hydration and local fluid volumes. A currently unanswered question is whether the relatively high “accelerated” air convection (and sublimed vapor transport) conditions present during both *in vitro* sublimation and release rate determinations, and *ex vivo* efficacy prolongation measurements, adequately model the intravaginal environment. The same question holds for the physiological validity of relatively low convection environments utilized during cell culture based efficacy prolongation experiments, and occurring intravaginally during testing in ewes, where excessive levels of viscous mucus were found to be continuously present. Therefore, all performance measures of subliming solid based drug delivery were, regardless of model employed, conducted in a relative manner, separately comparing structurally diverse drugs and then multiple subliming matrices to each other, thereby allowing the generalized conclusions discussed below.

### Drug Substance Release Rates from Subliming Matrices Were Independent of Active Agent Molecular Size and Structure

**Fig. 1:**
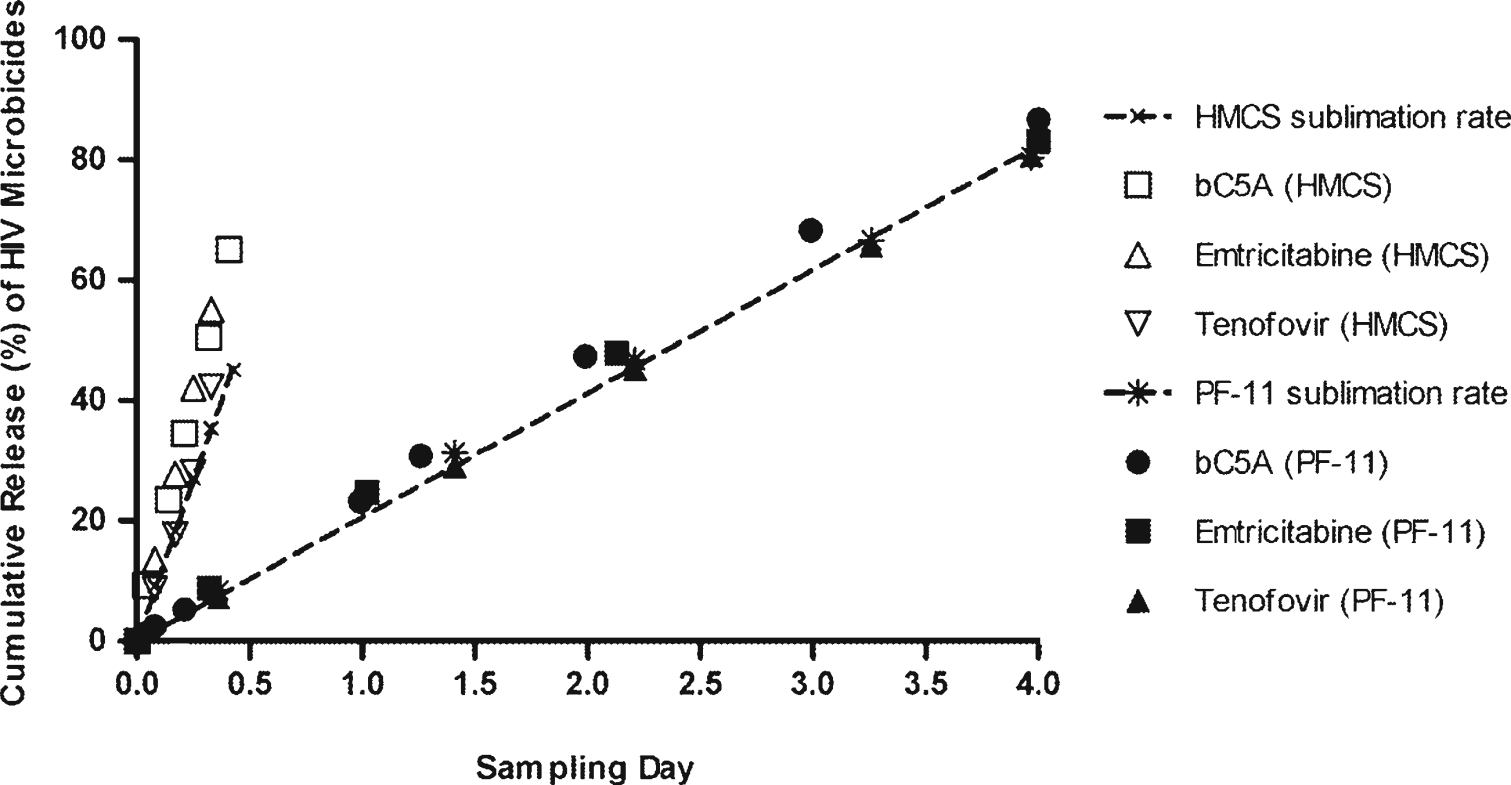
Comparison of *in vitro* (controlled convection) release rates of bC5A, emtricitabine and tenofovir, when incorporated within HMCS (*open symbols*) or PF-11 matrices (*filled symbols*). Each plotted point represents a mean ± SEM (*N* = 4).

Zero-order *in vitro* drug release from constant surface area matrix cylinders, measured by amounts of embedded microbicide particles progressively exposed on matrix pellet surfaces, appears to occur primarily through sublimation mediated surface erosion, with release rates of structurally dissimilar microbicide molecules being equal to the sublimation rate of the matrix into which they were incorporated, and independent of the drugs physicochemical properties (Fig. 1). Release rates of bC5A peptide, FTC and TDF were very similar to each other, in spite of their structural diversity and wide molecular size range, and equal to the sublimation rates of the HMCS or PF-11 matrices within which they were incorporated. This suggests that a particular duration of release was determined by the specific thermodynamic phase change properties of HMCS and PF-11 matrices providing sustained release.

Subliming matrix-based drug delivery compares favorably against existing diffusion/hydrolysis/dissolution based drug delivery technologies. Drugs of high molecular weight or hydrophilicity which typically diffuse through and release from water insoluble matrices very slowly (5), can instead be delivered from a given subliming solid at a rate independent of each potential agent’s structure and properties. Examination of Fig. 1 in particular demonstrates that i) release of a multi-charged but low aqueous solubility eighteen amino acid peptide (C5A) from a given matrix material occurs at a zero-order rate identical to those measured for much smaller TDF and much more water soluble FTC molecules, ii) that the release rates are identical to the intrinsic matrix monolith sublimation rate, and iii) that the equivalence of microbicide release and matrix sublimation rates holds true for several structurally and enthalpically different subliming solids.

Several existing processes for controlled delivery of drugs can also achieve zero-order release rates independent of active substance physicochemical attributes, entailing release of incorporated drug molecules/particles through the surface hydrolysis and/or dissolution of an entrapping matrix. Such release mechanisms are however often affected and sometimes controlled by local environment pH and hydration state (8–10). Neither of these variables should be a factor in the sublimation rates of the described hydrophobic matrix materials.

**Fig. 2:**
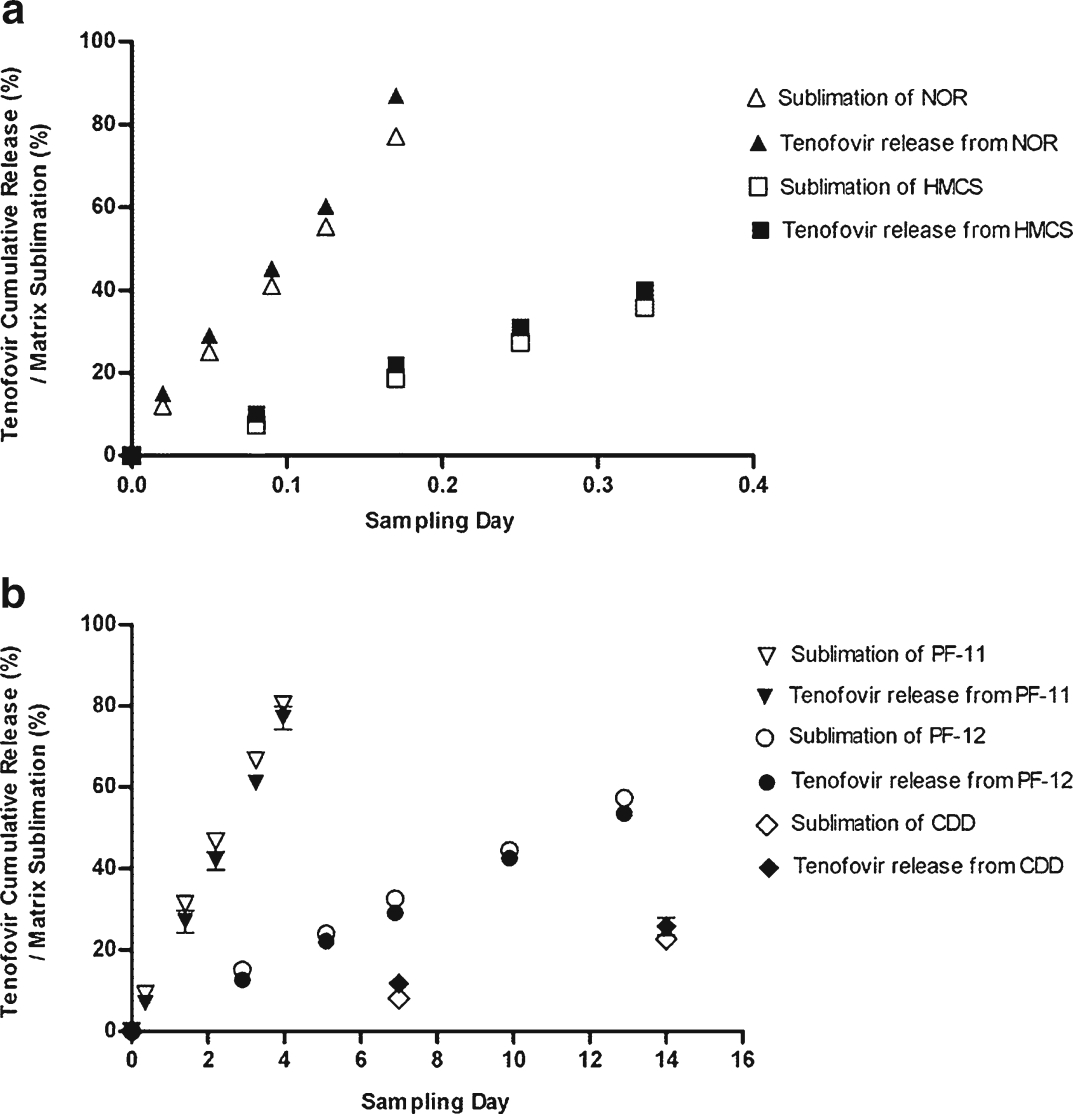
Comparison of tenofovir release rates *in vitro*, to matrix sublimation rates, for microbicide incorporated within a series of (**a**) rapidly subliming solids and (**b**) slowly subliming solids. Each plotted point represents a mean ± SEM (*N* = 4).

Use of subliming solids for drug delivery can also provide a broad available range of microbicide release rates and durations. Figure 2a and b demonstrates that under employed *in vitro* conditions, appropriate choice of matrix material can achieve constant rates of both matrix sublimation and concomitant tenofovir release over durations as short as hours (Fig. 2a) to as long as several months (Fig. 2b). Comparison of data represented by filled and open symbols in both Fig. 2a and b also conclusively demonstrates that the rate of microbicide exposure/release is identical to the rate at which a given matrix sublimes. This study has shown through the above *in vitro* demonstration of zero-order release for three dissimilar drug substances, incorporated into five different subliming matrices, that volatile water-insoluble organic solids hold promise as a multi month constant release rate delivery system for potentially any number of intravaginally administered HIV-1 microbicides.

**Fig. 3:**
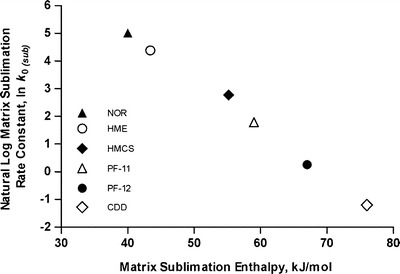
Natural log of matrix surface erosion rate constant (37°C, controlled convection) as a function of matrix material sublimation enthalpy. Each plotted point represents a mean value (*N* = 4).

The unprecedented range of release rates can be correlated to enthalpies of sublimation specific to each employed matrix material (Fig. 3), and allows a sublimation/release rate range of more than 6 (base e) log units. This correlation also suggests that the microbicide release mechanism is different from currently employed diffusion, dissolution, or hydrolytically mediated erosion processes for drug delivery. Sustained release of powdered drug substances through their incorporation into hydrophobic matrix monoliths where liberation of active agent occurs through sublimation driven surface erosion of matrix material, has therefore not previously been employed for drug delivery (26,27). Since the range of available sublimation rates is broad, the potential for short-term but continuous and controlled rectal delivery of HIV-1 microbicides also exists.

### Matrix Sublimation and Incorporated Drug Substance Release Rates Were Thermodynamically Controlled by Matrix Sublimation Enthalpy

The key intrinsic variable affecting the rate of matrix sublimation and concomitant drug release appears to be the sublimation enthalpy of the particular solid employed for sustained microbicide delivery. As sublimation enthalpies increased from 40 kJ/mol for NOR to 76 kJ/mol for CDD, a broad range of sublimation rates and equivalent release rates of incorporated TDF were achieved (Fig. 2a and b). The natural log of each erosion/release rate constant (mg/cm^2^/hr) was found to be a linear function of matrix sublimation enthalpy (Fig. 3) indicating thermodynamic control of the overall process, and explainable in several ways.

The physical process of sublimation can be described and quantitated employing transition state theory (28) where the molecular rate of sublimation is described by Eqs. (1) and (2), and where enthalpy of sublimation at body temperature is larger than entropy of sublimation.

1 $$ {\text{k}} = \left( {{{\text{k}}_{\text{B}}}{\text{T}}/{\text{h}}} \right){ \exp }\left( { - \Delta {{\text{H}}_{\text{sub}}}/{\text{RT}}} \right){ \exp }\left( {\Delta {{\text{S}}_{\text{sub}}}/{\text{R}}} \right) $$

2 $$ { \ln }\,{\text{k}}/{\text{T}} = - \Delta {{\text{H}}_{\text{sub}}}/{\text{RT}} + \Delta {{\text{S}}_{\text{sub}}}/{\text{R}} + { \ln }\,{{\text{k}}_{\text{B}}}/{\text{h}} $$

The process of matrix erosion can also be described by the Clausius-Clapyron equation (Eqs. 3 and 4), where the vapor pressure of gaseous sublimed material at a matrix surface is a function of temperature, total pressure at the subliming surface, and the sublimation enthalpy of the particular matrix material (29).

3 $$ {\text{dP}}/{\text{dT}} = {\text{P}}\Delta {{\text{H}}_{\text{sub}}}/{\text{R}}{{\text{T}}^{{2}}} $$

4 $$ { \ln }\,{\text{P}} = - \Delta {{\text{H}}_{\text{sub}}}/{\text{RT}} + {\text{c}} $$

Rate of matrix erosion (at a given temperature and pressure) is directly proportional to the achieved vapor pressure of sublimed molecules, and has been shown to be rate limiting (controlling) when either *in vitro* or *in vivo* removal of sublimed vapor by diffusive and convective processes is rapid relative to the rate at which individual molecules of the matrix escape the eroding surface (30). Similarly, the potential effect of variations in local temperature and pressure on matrix sublimation becomes a non-issue when drug release occurs within the body, where both mammalian temperature and internal pressure are maintained at constant values through homeostasis (31–33).

Net rates of sublimation based erosion *in vivo* can however be influenced by diffusion and convection processes which, post sublimation, remove gaseous or solvated matrix molecules from the site of administration, perhaps leading to fractal (but still linear) release kinetics (34,35). The potential retarding influence of mass transport at a given administration site (see below study in ewes) can however be compensated for by appropriate choice of matrix material, and/or use of specific sublimation rate enhancers.

### Intravaginal Matrix Sublimations Occurred at Constant and Thermodynamically Defined Rates

Accelerated *in vitro* microbicide release rate measurements entail sublimation of matrix surface directly into 37°C, 100% relative humidity air slowly convecting under defined and controlled conditions, and in direct communication with the atmosphere. An intravaginal environment on the other hand, while providing defined and homeostatically controlled temperature and internal pressure at the matrix surface, putatively entails additional mass transport of sublimed matrix vapors away from the eroding surface via a series of diffusion and convection steps typically observed for implanted devices (36,37). Adult ewes were chosen for an animal model due to their average intravaginal surface area being very similar to the average value measured in women (23,38,39). The human validity of this particular model is subject to the caveat that (what were judged to be) excessive amount of viscous mucus were encountered during the cylindrical matrix sublimation rate determinations described below.

**Fig. 4:**
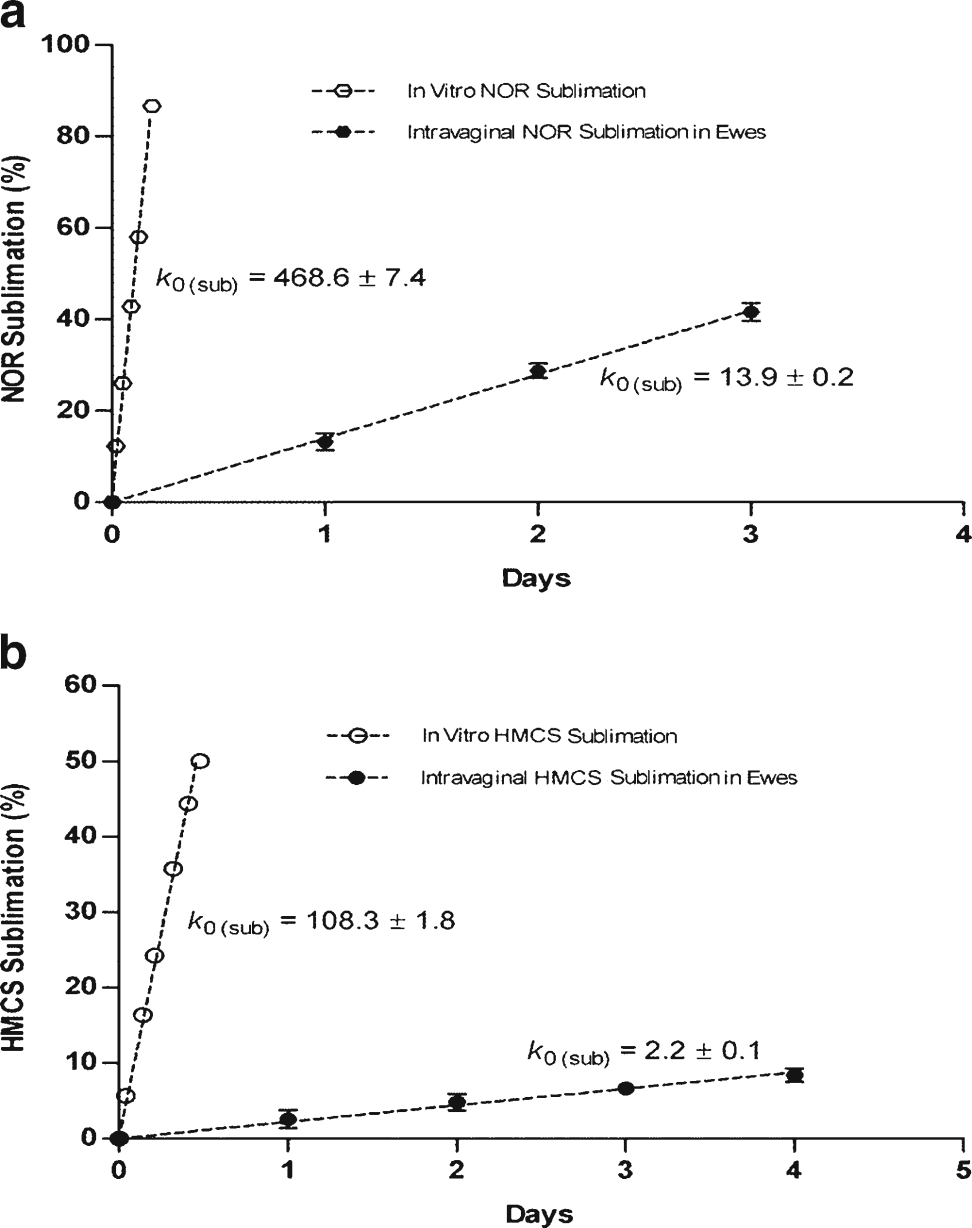
Comparison of intravaginal sublimation rates in ewes for (**a**) NOR and (**b**) HMCS matrices relative to “accelerated” rates obtained *in vitro* under controlled convection conditions. Each plotted point represents a mean ± SEM (*N* = 4).

For NOR and HMCS matrices, intravaginal sublimation occurred (similar to *in vitro* measurements) at zero-order (constant) rates (Fig. 4a and b), with the 4.5 fold *in vivo* difference in erosion rates between the two materials being similar if not equivalent to the 6.3 fold difference in rates measured using the “accelerated” *in vitro* model. The fact that the thermodynamically expected differences in sublimation rates between NOR and HMCS were measured both *in vitro* and *in vivo*, suggests that even for the most volatile of the evaluated matrices the thermodynamically controlled surface sublimation step remains rate limiting.

A requirement for ultimate success of any new drug delivery system is the ability to predictably function *in vivo*, and for clinically successful HIV microbicides, the ability to provide site specific delivery of adequate duration. We therefore showed that in the case of the above formulations/devices designed for intravaginal delivery of antiviral agents, differences in matrix-specific sublimation rates within ewes were very similar to the differences measured using the controlled convection “accelerated” *in vitro* method.

Demonstration of prolonged *in vivo* release achieving continuously virucidal concentrations of microbicides intravaginally, is planned as the primary follow-up activity to sheep and *ex vivo* experiments described in this study. Further studies will determine the locally-achieved concentration-based pharmacodynamics of leading HIV microbicides at the administration site, in macaques. More specifically, the agents will be intravaginally delivered at a series of constant rates, using subliming matrices incorporated into elastomeric vaginal rings (employed to help assure long term dose retention), with extent and duration of efficacy being determined through multiple viral challenges.

### Subliming Solid Matrices Were Nontoxic During Prolonged Contact with Human Peripheral Blood Monocytic Cells (PBMC cells), T-Lymphocytes, Macrophages and Ectocervical Explants

**Fig. 5:**
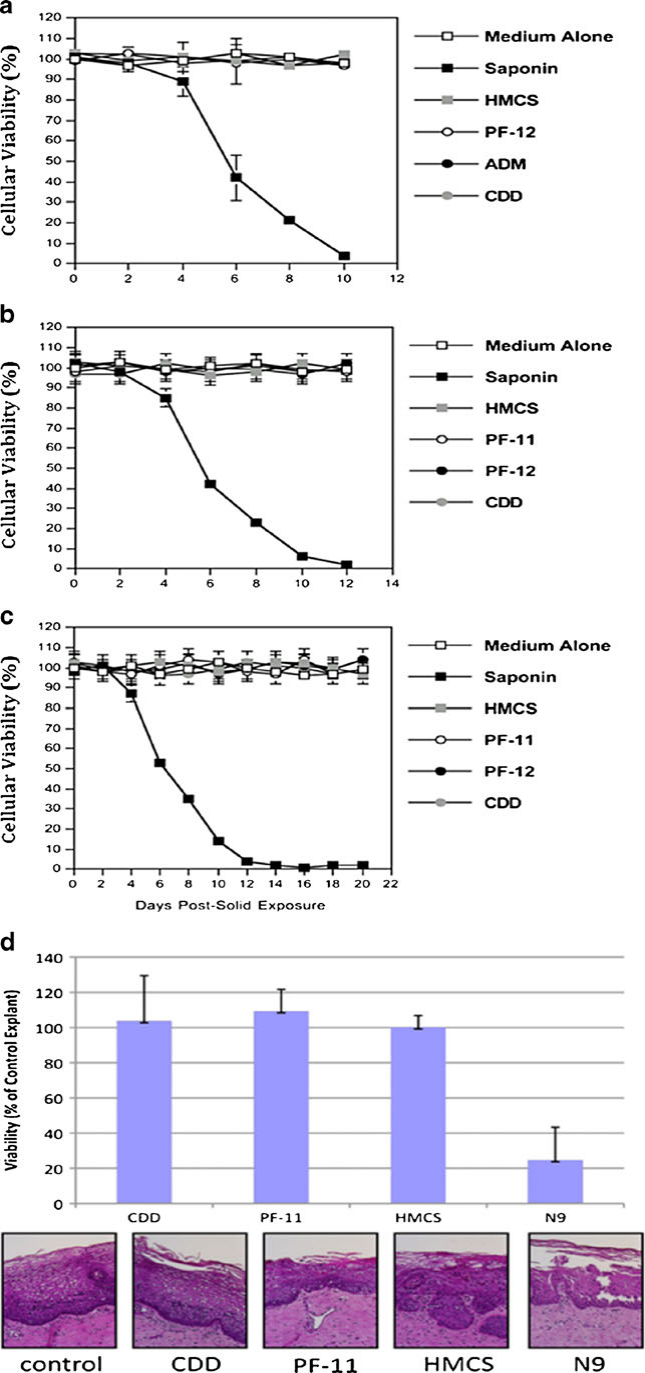
(**a**) Viability of human PBMC cells during 10 days exposure to HMCS, PF-12, ADM and CDD matrices. (**b**) Viability of cultured T-lymphocytes and (**c**) macrophages during 12 and 20 days exposures to HMCS, PF-11, PF-12, and CDD matrices. Data presented in (**a**)**,** (**b**) and (**c**) are expressed in percentage of viable cells. The error bars in (**a**), (**b**) and (**c**) represent standard errors of duplicates. Data shown in panels (**a**), (**b**) and (**c**) are each representative of two independent experiments. (**d**) *Ex vivo* viability of human ectocervical tissue during 5 days exposure to CDD, PF-11, and HMCS matrices. The data represent the mean ± SD of three independent tissues performed in duplicate. The viability of the N9-treated tissues were significantly reduced from control (untreated) tissues (*P* < 0.05) (Wilcoxon *T*-test).

Subliming solid matrix materials under investigation appear to have low toxicity and acceptable biocompatibility. Human PBMC cell culture remained 100% viable after 10 days exposure to partially immersed HMCS, PF-12, ADM, and CDD matrix discs (Fig. 5a). Other intravaginal HIV-1 target cells such as human T-lymphocytes (Fig. 5b) and macrophages (Fig. 5c) exhibited full viabilities over 12 and 20 day periods of continuous exposure to HMCS, PF-11, PF-12, and CDD pellets. A saponin control demonstrated intrinsic sensitivity of each cell type to toxic substances. Viability of ectocervical tissue after 5 days continuous exposure to the different subliming matrix discs did not differ from control tissue cultured in parallel (Fig. 5d). Nonoxonyl-9 (N9) served as a toxic substance control. In summary, no evidence of sub-acute toxicity was observed with any of the matrix materials, whether evaluated in ectocervical tissue, macrophages, T-lymphocytes, or PBMC. This very preliminary indication of low toxicity for our delivery system provides the basis for more extensive future studies evaluating local irritation potential, and chronic toxicology assessment intravaginally and systemically. The ultimate clinical viability of sustained intravaginal microbicide release in general, and the potentially long-term intravaginal exposure to vaporized subliming solids, will require demonstration of acceptable drug product toxicology on a case by case basis for each particular microbicide plus subliming matrix combination.

### Release of Drugs from Subliming Matrix Prolonged Antiviral Activity Measured in Human Ectocervical Explants

**Fig. 6:**
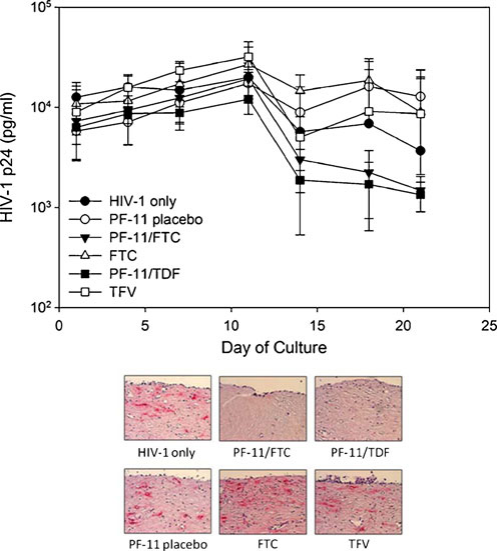
Effect of emtricitabine (FTC) and tenofovir disoproxil fumarate (TDF) incorporated into PF-11 matrix pellets on HIV infection in ectocervical explants. HIV-1 infection is monitored over 21 days by HIV-1 p24gag protein detected in the basolateral culture supernatant. Endpoint immunohistochemistry was performed to confirm infection. The presence of infected cells (red deposits) is indicative of p24-positive cells. The data presented are the median ±95% confidence interval of three independent tissues and representative immunohistochemistry figures.

To be a therapeutically relevant drug delivery system, especially in the context of extended duration microbicide delivery, drug loaded subliming matrices should provide prolonged and continuous antiviral activities. A clinically relevant *ex vivo* model for sexual infection examining residual infectivity subsequent to multiple HIV challenge of human extocervical explants, was used to demonstrate this capability. A result typical of multiple challenge is for HIV-1 p24 concentrations to initially rise, then decrease upon challenge cessation, and then continue to drop to a steady value indicating the extent of residual infection (Fig. 6). The antiviral activity of microbicide candidates is determined by whether the tissues are protected against infection, relative to untreated controls. An advantage to using ectocervical explants for product testing is that appropriate HIV-1 target cells are present in the appropriate ratio. Using this model (23,24), we determined whether a given subliming solid is capable of delivering sufficient amounts of TDF or FTC to the tissue and prevent HIV-1 residual infection after multiple viral challenge. Over the course of the 21 day culture period, viral challenge occurred every 3 to 4 days over the first 10 days of culture (4 independent exposures to HIV-1) with treatment entailing either a single bolus administration of TDF or FTC in solution prior to the first challenge, or continuous release of TDF or FTC from PF-11 matrix discs during and subsequent to challenges. After virus challenges ceased, HIV-1 p24 levels decreased in the control explants, in those treated with either TDF or FTC in solution, and those treated with placebo matrices, to a constant level of 3.8 log_10_ pg/mL by day 17 of culture (Fig. 6).

The TDF/PF-11 and FTC/PF-11 treated explants continued to show further reduction in HIV-1 p24 concentrations out to 21 days, indicating termination of infection. This was confirmed by immunohistochemistry which showed absence of HIV-1 infected cells only in explants which continuously received TDF or FTC. This is in contrast to explants treated with an unformulated TFV or FTC single dose, or those serving as controls, all of which showed no protection against residual infection (Fig. 6) as confirmed by high levels of p24 (3.6 log_10_) still being measured at day 21, and presence of p24-positive cells (pink) visualized by immunohistochemistry.

### Release of the Three Dissimilar Drugs from Several Subliming Matrices Prolongs Antiviral Activities Measured in Human Macrophages and TZM Reporter Cells

**Fig. 7:**
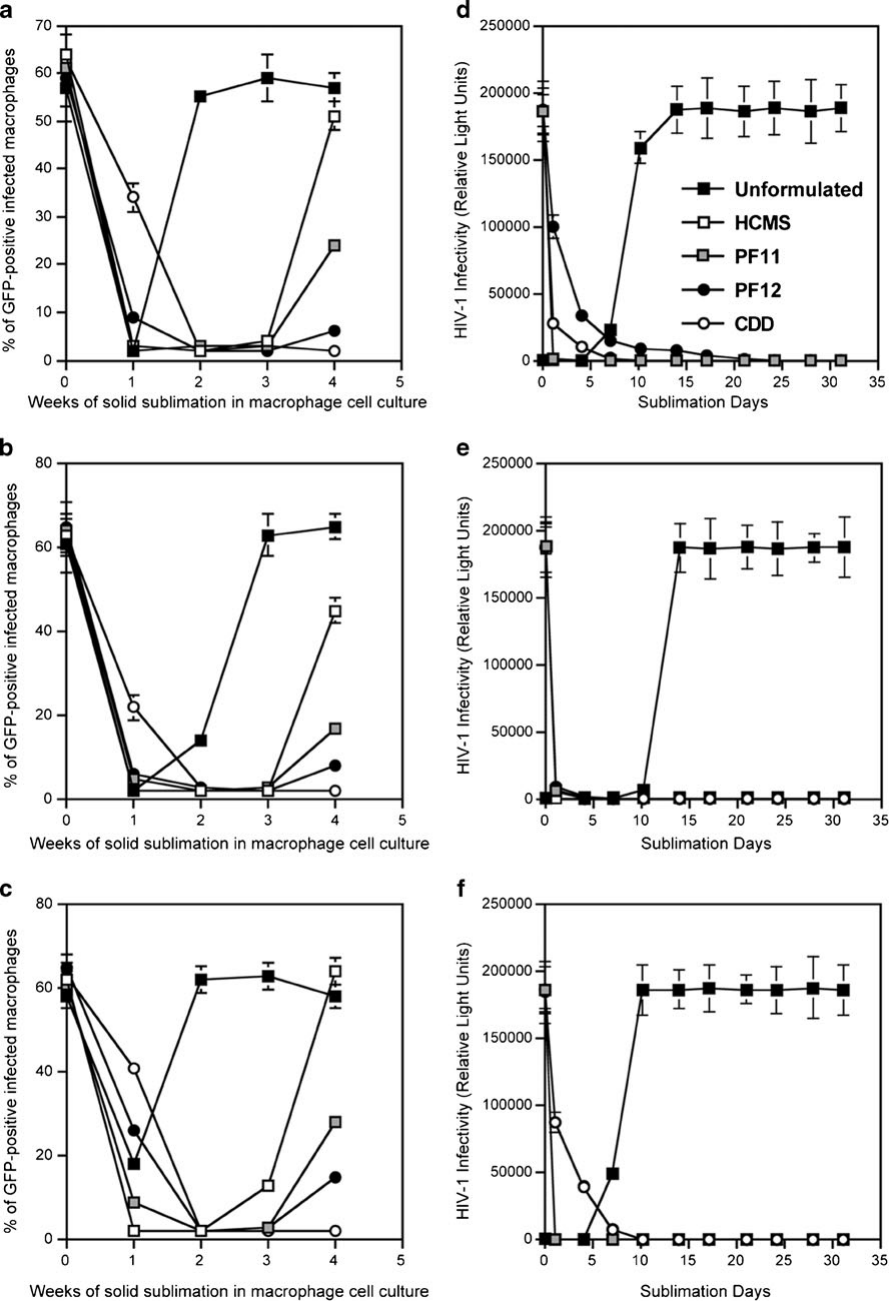
Anti-HIV-1 activity prolongation of (**a**) tenofovir, (**b**) emtricitabine, and (**c**) bC5A in human macrophage cell culture during delivery from HMCS, PF-11, PF-12, and CDD matrices. Antiviral activity prolongation in TZM reporter cells due to release of (**d**) tenofovir, (**e**) emtricitabine, and (**f**) bC5A from HMCS, PF-11, PF-12, and CDD matrices. The error bars represent standard errors of triplicates.

To further demonstrate the ability of subliming solid based delivery to enhance and prolong antiviral activity of TDF, bC5A, and FTC, durations of protection against HIV-1 challenge were determined using human macrophages and TZM reporter cell cultures (40), maintained in a low convection environment to provide an alternate approximation of the vaginal interior. The panels in Fig. 7a-c show that for macrophages infected with HIV-1 (GFP), complete protection by all three microbicides was maintained for at least 3 weeks, regardless of whether the drug incorporating matrix material was HMCS, PF-11, PF-12, or CDD. However, by the fourth week only the microbicides incorporated into slowest subliming matrix (CDD) were still providing complete protection, with durations of residual protection provided by the other matrices being inversely proportional to their intrinsic rates of sublimation (Fig. 2a and b). In comparison, unformulated microbicides (aqueous solutions in culture medium) in all cases provided fewer than 2 weeks of complete protection. Anti-HIV-1 activity prolongation experiments employing TZM reporter cells (Fig. 7d-f), cultured under the same low convection conditions, appeared to show complete elimination of viral infection (at least out to 30 days) regardless of whether the microbicide was TDF, FTC or bC5A, when released from any of the four matrix materials. Aqueous solutions of the microbicides on the other hand could not prevent re-establishment of infections, which in all cases occurred after only 7–12 days of continuous incubation.

To summarize, physicochemical and preliminary safety evaluations of this delivery system correctly predicted the benefits of subliming solids for prolongation of anti-HIV effect during multiple viral challenge, as demonstrated *ex vivo* for TDF and FTC released from PF-11 pellets onto ectocervical explants. The ability of all three matrix incorporated microbicides to protect both macrophages and TZM reporter cells from a high dose viral challenge, even subsequent to their continuous sublimation and release for several weeks, confirms the therapeutic potential of sublimation based drug delivery for HIV prophylaxis prolongation. Sustained and controlled delivery of a potentially large variety of active agents through the sublimation of surrounding matrix may shift the microbicide-based prevention of sexual HIV transmission paradigm from one where mode and mechanism of delivery (the formulation) is secondary to the biological properties of the specific microbicide drug substance, to one where sustained unobtrusive intravaginal delivery will be essential for, and successfully employed in, a majority of future prophylactic vaginal microbicide products.

## CONCLUSION

Providing a predetermined and broad range of release rates and durations for a variety of structurally dissimilar HIV-1 drugs in a matrix enthalpy dependent manner is the first step towards demonstration and development of an unprecedented delivery system. Subliming solid based matrices appear to be readily applicable to controlled release of many current and future microbicide candidates where continuous and extended duration administration would be therapeutically beneficial, regardless of their molecular size, structure, or physiochemical properties. A perhaps universal drug delivery capability for implantable systems including intravaginal rings has therefore been identified and is being actively investigated.
